# Distribution, biosynthesis, and synthetic biology of phenylethanoid glycosides in the order Lamiales

**DOI:** 10.5511/plantbiotechnology.24.0720a

**Published:** 2024-09-25

**Authors:** Yushiro Fuji, Hiroshi Matsufuji, Masami Yokota Hirai

**Affiliations:** 1 RIKEN Center for Sustainable Resource Science, Yokohama, Kanagawa 230-0045, Japan; 2 Department of Food Science and Technology, College of Bioresource Sciences, Nihon University, Fujisawa, Kanagawa 252-0880, Japan; 3 Department of Applied Biosciences, Graduate School of Bioagricultural Sciences, Nagoya University, Nagoya, Aichi 464-8601, Japan

**Keywords:** acteoside, biosynthesis, Lamiales-order, mass production, phenylethanoid glycoside

## Abstract

Phenylethanoid glycosides (PhGs), with a C_6_-C_2_ glucoside unit as the basic skeleton, are specialized (secondary) metabolites found in several medicinal plants. As PhGs exhibit various pharmacological activities, they are expected to be used as lead compounds in drug discovery. However, mass-production systems have not yet been established even for acteoside, a typical PhG that is widely distributed in nature (more than 150 species). This review focuses on recent studies on the accumulation and distribution of PhGs in plants, biosynthetic pathways of PhGs, and the bioproduction of PhGs.

## Introduction

**Figure 1:**
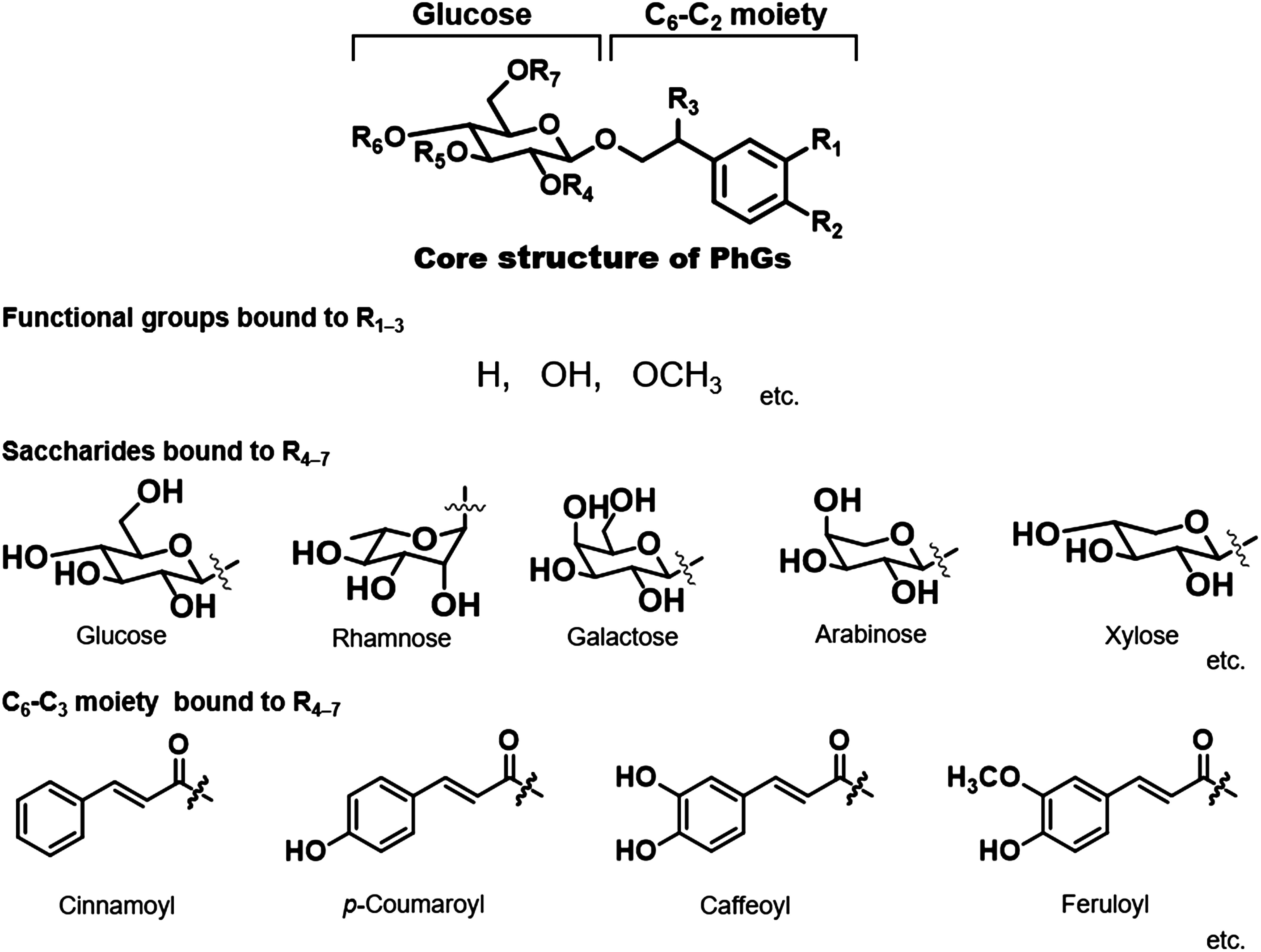
Figure 1. Basic structure of phenylethanoid glycoside and frequently occurring substituents.

Phenylethanoid glycosides (PhGs) are natural products mainly derived from plants of the order Lamiales and have been widely used as traditional medicines worldwide (Alipieva et al. 2014). The core structure of PhGs consists of a C_6_-C_2_ unit with β-glucopyranose (β-glucose) linked by a glycosidic bond (Figure 1). It is often abundantly decorated with substituents such as aromatic acids (e.g., cinnamic acid, coumaric acid, caffeic acid, and ferulic acid) and various saccharides (e.g., glucose, rhamnose, galactose, and arabinose) through ester or glycosidic bonds (Xue and Yang 2016). In recent years, there has been increasing interest in PhGs, and a significant increase in reports on the pharmacological effects of PhGs, their preventive and therapeutic effects on various diseases, and their pharmacokinetics.

Acteoside, also known as kusagin or verbascoside, is the most common PhG and has been reported in over 150 plant species belonging to 20 families and 77 genera of mainly the order Lamiales (He et al. 2011). Acteoside has been studied extensively for its various pharmacological activities including antioxidative (Luhata and Usuki 2022), anti-inflammatory (Kahraman et al. 2022; Seo et al. 2013; Zhou et al. 2021), hepatoprotective (Jaramillo-Morales et al. 2023; Jing et al. 2015), and neuroprotective (Jia et al. 2020; Koo et al. 2005) activities. Previous studies have also shown the potential of acteoside as a therapeutic compound for neurodegenerative diseases such as Alzheimer’s (Chen et al. 2022; Korshavn et al. 2015; Shiao et al. 2017) and Parkinson’s disease (Aimaiti et al. 2021; Yuan et al. 2016). Moreover, due to its low side effects, acteoside has the potential to develop into a promising pharmaceutical agent, which has attracted attention in recent years. To date, acteoside has been prepared through three main approaches: isolation from plants, chemical synthesis, and biosynthesis using heterologous host.

In this article, we focused on mainly acteoside as a representative PhG and provide an overview of its accumulation sites and biosynthetic pathways in Lamiales plants, and synthetic biology utilizing microorganisms.

## Major phenylethanoid glycosides in nature

**Figure 2:**
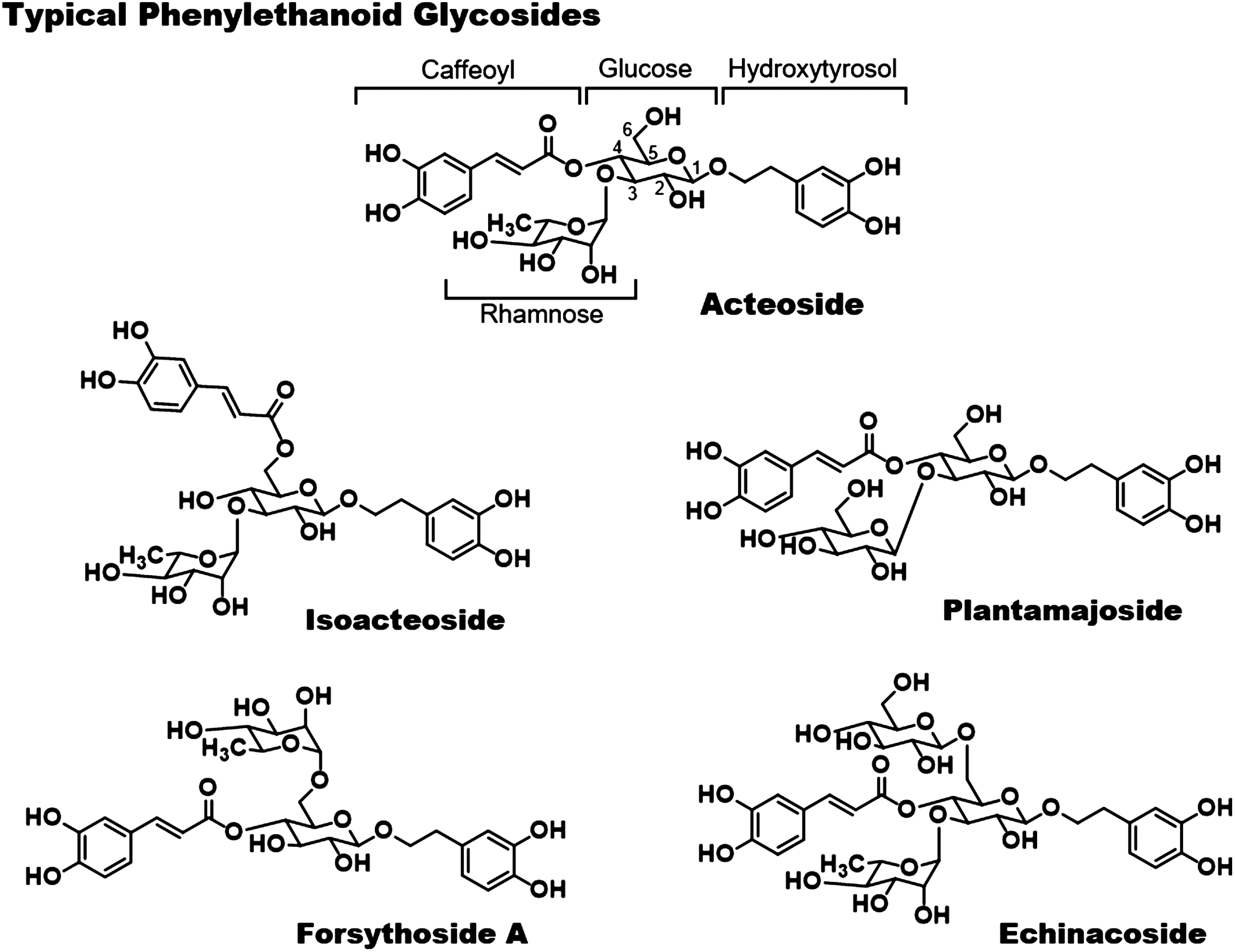
Figure 2. Typical structures of phenylethanoid glycosides.

The typical compounds of PhGs are shown in Figure 2. Acteoside, in which rhamnose attached to the 3-position of the glucose unit of hydroxytyrosol-1-*O*-glucoside and caffeic acid attached to the 4-position, is the most representative PhGs. It is widely distributed among more than 150 species of various medicinal plants belonging to 20 families and 77 genera, including *Conandron ramondioides*, *Proboscidea louisiana*, *Leucosceptrum japonicum*, *Cistanchis* species (*Cistanche deserticola*, *Cistanche tubulosa*, *Cistanche salsa*, and *Cistanche sinensis*), *Stachys sieboldii*, *Plantago asiatica*, *Plantago depressa*, *Rehmanniae radix*, *Olea europaea*, etc (He et al. 2011). On the other hand, isoacteoside is an isomer of acteoside that differs in the bonding position of caffeic acid. Isoacteoside is often found together in plants containing acteoside, but the amount of isoacteoside is small compared to the amount of acteoside present. Plantamajoside in which the rhamnose moiety of the acteoside is replaced by glucose is one of the main active ingredients in *P. asiatica* (Ravn et al. 2015). Forsythoside A is a PhG formed from hydroxytyrosol 1-*O*-glucoside, with caffeic acid and rhamnose attached to the C-4 and C-6 of glucose, respectively and is found in *Forsythia suspensa* (Wang et al. 2018). Echinacoside is the oldest among the known PhGs, having been found in *Echinacea angustifolia* in 1950. The structure of echinacoside, which is a trisaccharide glycoside consisting of an additional glucose bonded to the glucose 6 position of acteoside, was determined in 1982, and it is abundant in *E. angustifolia* and *C. tubulosa*. Echinacoside is found in more than 40 species of medicinal plants belonging to 10 families and 18 genera (Liu J et al. 2018).

**Figure 3:**
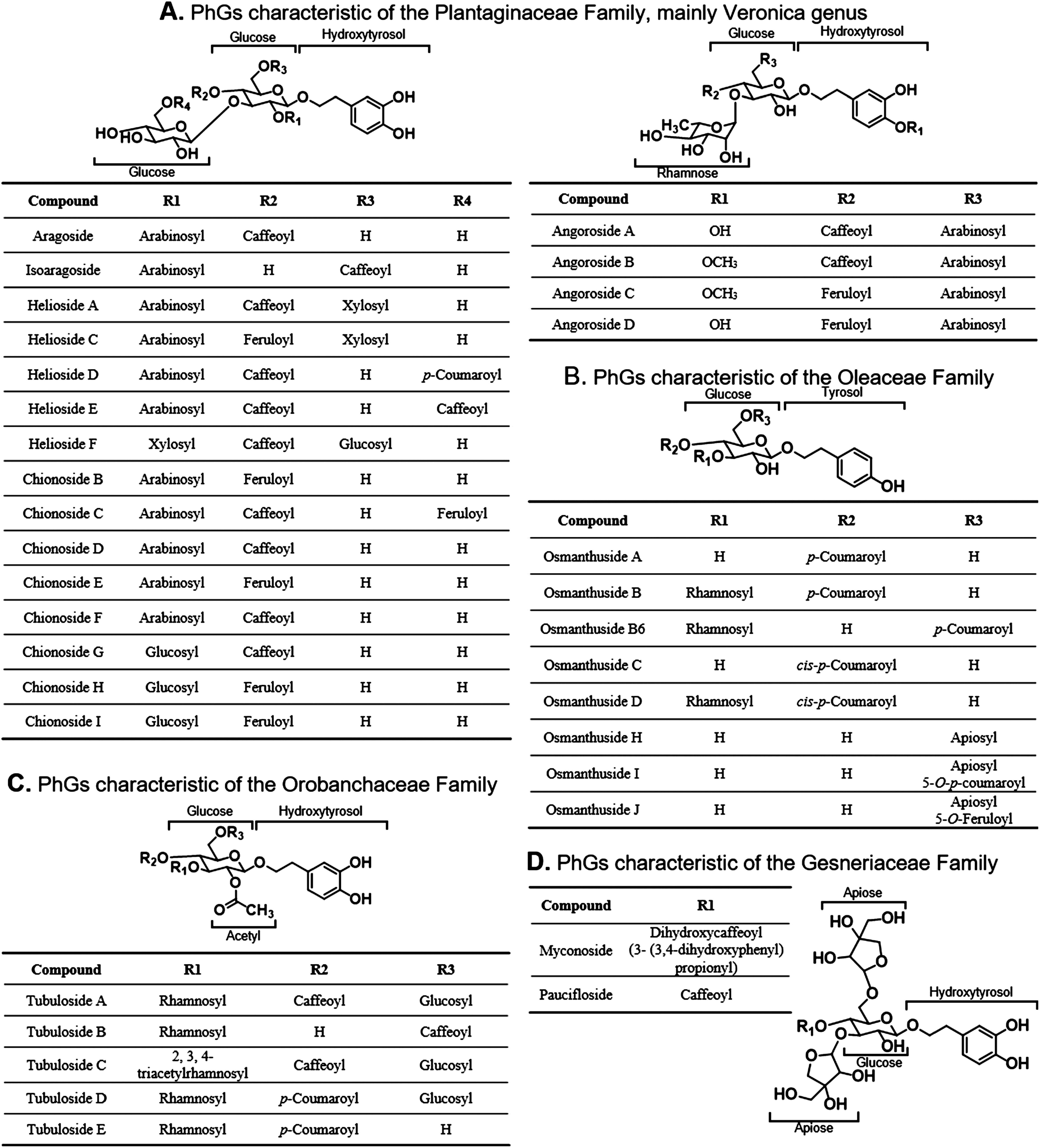
Figure 3. Structure of family-specific phenylethanoid glycosides in the order Lamiales. (A) Chemical structures of the Plantaginaceae family; (B) Chemical structures of the Oleaceae family; (C) Chemical structures of the Orobanchaceae family; (D) Chemical structures of the Gesneriaceae family.

Among the family-specific PhGs in the order Lamiales, Plantaginaceae family plants contain a variety of PhGs such as chinoside, aragoside, and helioside shown in Figure 3A, in which arabinose is attached to the 2-position of glucose (Rønsteda et al. 2003; Taskova et al. 2010, 2012). Additionally, angoroside, in which arabinose is attached to the 6-position, has also been isolated from plants of the Plantaginaceae family (Calis et al. 1988; Miyase and Mimatsu 1999; Wang S et al. 2017). Heliosides (A–F), which are found in *Veronica* species and widely distributed in New Zealand, could serve as markers for identifying these species (Taskova et al. 2012). Plants of the Oleaceae family are distributed in temperate and tropical regions and are classified into genera such as *Osmanthus*, *Syringa*, and *Ligustrum* based on the shape of their flowers, leaves, and fruits. Osmanthuside (Figure 3B) is a common PhG found in the Oleaceae family, mainly composed of salidroside (β-(*p*-hydroxyphenyl)ethyl β-D-glucopyranoside), *p*-coumaric acid, and rhamnose. It is a major component in *Osmanthus asiaticus* and *Ligustrum robustum* (He et al. 2003; Sugiyama and Kikuchi 1990). Tubuloside (Figure 3C), which has an acetyl group at the 2-position of glucose, is a characteristic PhG isolated from *Cistanche*, a traditional Chinese medicine plant in the Orobanchaceae family (Kobayashi et al. 1987; Yoshizawa et al. 1990). *Haberlea rhodopensis* and *Sanango racemosum* belong to the Gesneriaceae family and contain myconoside and paucifloside, which have the structure with apiosyl moieties attached to C-3 and C-6 of glucose (Figure 3D; Jensen 1996; Zheleva-Dimitrova et al. 2016).

On the other hand, although not in the order Lamiales, Magnoloside (A–P), which are rarely found in the plant kingdom, have been isolated from plants in the order Magnoliales, such as Magnolia (*Magnolia officinalis*, *Magnolia obovata*). In magnolosides, the glucose of core structure is replaced by allopyranose (Ge et al. 2017; Xue et al. 2016; Yu et al. 2012). Although there are reports of the detection of acteoside and isoacteoside in Magnolia (Porter et al. 2015), the determination is questionable because it is based on chromatographic and mass spectrometric comparison with a standard and does not distinguish between glucopyranose and allopyranose.

As mentioned above, PhGs have many similar structures, and numerous PhGs are present in plants. For example, *C. tubulosa*, which is rich in echinacoside and acteoside, contains as many as 26 minor PhGs (Morikawa et al. 2019). Therefore, PhGs have attracted attention as a chemotaxonomic indicators, which attempts to classify plant phylogeny based on the composition and structure of these plant specialized metabolites and their presumed biosynthetic pathways.

## Localization and accumulation of acteoside and other PhGs in plants

Acteoside has been detected in both the underground and above-ground parts of plants, but at widely varying levels. Olive (*O. europaea*) is one of the most studied acteoside-containing plants. Ryan et al. (2003) monitored the phenolic profiles of the pulp, seed, stone, and leaves of *O. europaea* during two fruiting periods. They reported that hydroxytyrosol, a precursor of acteoside, accumulated predominantly in the leaves (approximately 127 mg g^−1^ dry weight (DW) tyrosol equivalent), whereas hydroxytyrosol glucoside was detected only in mature fruits, although the accuracy of quantitation is limited as they assumed that all compounds have the same response coefficient as tyrosol and quantified them using the tyrosol calibration curve. In contrast, acteoside was the most abundant in the pulp (approximately 17 mg g^−1^ DW tyrosol equivalent) but was not detected in the leaves. Alagna et al. (2012) studied the acteoside content in the developing fruits of 12 olive cultivars and reported that Coratina and Rosciola varieties contained up to 9.0% acteoside per dry fruit, at 90 days after flowering, whereas other varieties had less than 3.0%. Other plants, such as *Verbascum nigrum* (Leaves, 3.03%) and *Verbascum xanthophoenicem* (Leaves, 1.58%) (Georgiev et al. 2011), *C. tubulosa* (Stem, 0.86–2.54%), *Cistanche desericola* (Stem, 0.86–2.54%), *C. deserticola* (Stem, 0.48–2.11%) (Shi et al. 2009; Wu et al. 2006) and *P. lanceolata* (Aerial parts, 2.17%) (Janković et al. 2012) have been reported as acteoside-rich plants. *Aloysia citriodora*, also known as lemon verbena, is a medicinal plant rich in acteoside, with leaves reportedly containing 0.5–4.8% of acteoside (Lira et al. 2010). Leaf extracts have long been used in traditional medicine, e.g., in European and North American, as well as traditional Chinese medicine. On the other hand, in previous studies (Fuji et al. 2018a, 2018b), we examined the accumulation of acteoside during growth and in different organs of the sesame plant (*Sesamum indicum*). The study found that the acteoside content was increased during growth, reaching a maximum of 12.9% in dry leaves during the flowering period. The highest acteoside content was found in the leaf blades (12.3% DW), followed by petioles (3.1%) and petals (2.7%), while it was either present at trace levels or not detected in other organs. This is one of the highest levels reported in plants in nature. Sesame is found to be a rare plant that produces high levels of acteoside.

For other PhGs, major molecular species differed among families even within the Lamiales order. Myconoside (Figure 3D) was found to be the dominant compound in the *H. rhodopensis* extract (332.2±0.7 mg g^−1^ DW), remarkably reaching 88.8% of leaf extract (Zheleva-Dimitrova et al. 2016). *Cistanche* species (including *C. deserticola*, *C. tubulos*a, *C. salsa*, and *C. sinensis*) are parasitic plants belonging to the Orobanchaceae family that have long been used in traditional Chinese medicine. The major PhGs vary depending on the species. In the stolon of *C. salsa*, acteoside, echinacoside, and poliumoside (the rhamnoside of acteoside) were found to be the main compounds (9.44±0.01, 10.98±0.01, and 5.68±0.01 mg g^−1^ DW, respectively) (Kartbaeva et al. 2017). Trampetti et al. (2019) analyzed the flowers, stems, and roots of *Cistanche phelypaea* and found that acteoside was predominantly detected in the stems, while echinacoside and tubuloside A were mainly found in the roots. Piwowarczyk et al. (2020) elucidated the polyphenol profile of *Cistanche armena* when parasitizing *Alhagi maurorum* (a plant of the Fabaceae family plant) and *Salsola dendroides* (Amaranthaceae family plant) as hosts. Acteoside, echinacoside, and β-hydroxyacteoside are the major PhGs in *C. armena*. In the flowers of *C. armena*, echinacoside was detected at levels of 0.16 mg g^−1^ (when parasitizing *S. dendroides*) and 0.29 mg g^−1^ (when parasitizing *A. maurorum*). However, echinacoside was not detected in the stem with tuber. Interestingly, in the stems of *C. armena* parasitizing *S. dendroides*, the levels of acteoside and β-hydroxyacteoside were significantly higher at 7.41 and 10.7 mg g^−1^ DW, respectively, compared to 2.85 and 1.93 mg g^−1^ DW when parasitizing *A. maurorum*, indicating significant variations depending on the host (Piwowarczyk et al. 2020). Soares et al. (2020) compared the levels of three bioactive caffeic acid derivatives (plantamajoside, iso-plantamajoside, and acteoside) in the leaves of three *Plantago* species (*Plantago major*, *Plantago media*, *Plantago lanceolata*) and three varieties of *P. major*, cultivated in greenhouse and outdoor environments. They found that in *P. major*, both during greenhouse and outdoor cultivation, plantamajoside was more abundant than acteoside. However, in *P. media* and *P. lanceolata*, the opposite result was observed. They reported that plants cultivated in a greenhouse, where UV radiation was lower, had lower levels of all bioactive caffeic acid derivatives than plants grown outodoors. Murai et al. (2009) investigated in the amount of UV-absorbing compounds in *P. asiatica* and detected both plantamajoside and acteoside in the leaves. However, acteoside was not detected in plants from lower elevation areas with lower UV intensities. Li et al. (2016) also revealed that the amount of plantamajoside and the yield of *P. depressa* were significantly influenced by the amount of water supplied during the cultivation period. They reported a positive correlation between the plantamajoside level, net photosynthetic rate, and C/N ratio. These results indicate the induction of PhGs production by UV radiation and the role for PhGs in UV protection in plants.

The PhGs of Forsythia species belonging to the Oleaceae family has been well studied by Zürn et al. (2021) and is reported to be characterised by forsythoside A, as discussed in detail below. In *Forsythia europaea*, chemical diversity has been observed among plants from different habitats. The main PhGs compound in Far Eastern leaves and fruits is forsythoside A, whereas the main PhGs compound in European leaves is acteoside. In Hungarian *F. europaea* leaves and fruits, acteoside was confirmed as the main compound. The hybrid plant *Forsythia*×*intermedia* has several well-defined cultivars, and its leaf compounds have been analyzed due to the absence of fruit formation in temperate climates. Forsythoside A is the main component of *F.×intermedia* cultivars rather than acteoside (Zürn et al. 2021). In *F. suspensa*, forsythoside A was the main compound in both the fruits and leaves (88.3 mg g^−1^ DW). Isolated seeds from unripe fruits were rich in forsythoside A (47 mg g^−1^ DW). The compositional analysis of the fruit parts during ripening revealed that unripe fruit walls accumulated high amounts of acteoside in *F. europaea* and forsythoside A in *F. suspensa*. The unripe fruit walls in *F. europaea* and *F. suspensa* accumulated large amounts of acteoside (71.4 mg g^−1^ DW) and forsythoside A (80.4 mg g^−1^ DW), respectively. The amount of these PhGs were negligible in the walls of ripe fruits, confirming that the decomposition of acteoside and forsythoside A takes place in the fruit walls during the ripening process (Zürn et al. 2021).

As described above, although the main PhGs differed among the families, basically acteoside is also detected as the main component. On the other hand, it is very interesting to note that the accumulation sites of acteoside and PhGs in which acteoside is further modified with sugar or acetyl groups are different. Further studies are needed on the biosynthetic pathway of the modified enzymes after acteoside and on the differences in physiological functions in plants.

## Acteoside biosynthesis

**Figure 4:**
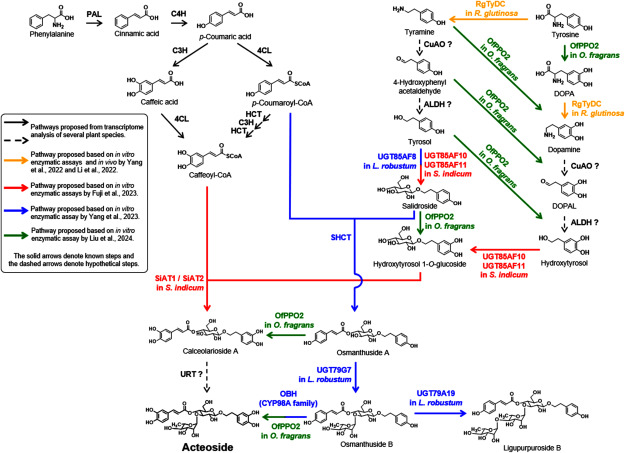
Figure 4. Proposed acteoside biosynthetic pathway. The solid arrow represents the known steps, and the dashed arrows denote the putative steps. Black arrows indicate the proposed pathways from transcriptome analysis of several plant species, yellow arrows indicate the proposed pathway based on the in vitro enzymatic assay and in vivo in *Rehmannia glutinosa* by Yang et al. (2022) and Li et al. (2022), red arrows indicate the proposed pathway based on the in vitro enzymatic assay in *Sesamum indicum* by Fuji et al. (2023), blue arrows indicate the proposed pathway based on the in vitro enzymatic assay in *Ligustrum robustum* by Yang et al.(2023), and green arrows indicate the proposed pathway based on the in vitro enzymatic assay in *Osmanthus fragrans* by Liu et al. (2024). Enzyme abbreviation: PAL, phenylalanine ammonia-lyase; C4H, cinnamate-4-hydroxylase; C3H, coumarate-3-hydroxylase; 4CL, 4-coumoyl-CoA ligase; HCT, 4-hydroxycinnamoyl CoA:shikimate/quinate hydroxycinnamoyltransferase; TyDC, tyrosine decarboxylase; CuAO, copper-containing amine oxidase; ALDH, alcohol dehydrogenase; PPO, polyphenol oxidase; CYP, cytochrome P450; URT, UDP-rhamnose glycosyltransferase; AT, acyltransferase; SHCT, hydroxycinnamoyl-CoA:salidroside hydroxycinnamoyltransferase; OBH, osmanthuside B hydroxylase.

The research on the acteoside biosynthetic pathway began more than 50 years ago. Isotope labeling experiments using lilac (Ellis 1988) and olive cultured cells (Saimaru and Orihara 2010) have revealed acteoside precursors and presumed an acteoside biosynthetic pathway. These researches have revealed the caffeoyl moiety of acteoside is biosynthesized from phenylalanine via cinnamic and *p*-coumaric acids, whereas hydroxytyrosol moiety is biosynthesized from tyrosine via tyramine and dopamine as intermediates. Subsequently, transcriptome analysis of *O. europeae* (Alagna et al. 2012), *Rehmannia glutinosa* (Wang F et al. 2017), and *Forsythia* species (Sun et al. 2018) led to the selection of candidate enzyme genes in the putative pathway. As described in more detail below, recent studies (Fuji et al. 2023; Yang et al. 2023) have also revealed enzymes responsible for glycosylation and acylation that are important for the formation of the acteoside skeleton by enzymatic assays. The biosynthetic pathway proposed on the basis of previous acteoside biosynthesis research is shown in Figure 4. The phenylpropanoid pathway from phenylalanine to caffeoyl-CoA is common in flavonoid and lignin biosynthesis, and the enzyme genes involved in this pathway have been extensively characterized in various plant species (Vanholme et al. 2019; Winkel-Shirley 2001). On the other hand, genes encoding the enzymes responsible for the formation of hydroxytyrosol from tyrosine [namely, copper-containing amine oxidase (CuAO), and alcohol dehydrogenase (ALDH)] shown as dashed arrows in Figure 4, have been proposed to be involved in the acteoside biosynthetic pathway based on transcriptome analysis, but have not been identified (Alagna et al. 2012; Sun et al. 2018; Wang F et al. 2017). As indicated by the yellow arrows in Figure 4, the in vitro and in vivo enzyme activity of tyrosine decarboxylase (TyDC) has been reported in *R. glutinosa*, suggesting its involvement in acteoside biosynthesis (Li et al. 2022; Yang et al. 2022). On the other hand, the most recent findings of acteoside biosynthetic research were reported by Liu et al. (2024) on PPO in *Osmanthus fragrans* (OfPPO2, green arrow in Figure 4). Liu et al. demonstrated that OfPPO2 hydroxylates various monophenolic substrates to generate catechol-containing intermediates essential for acteoside biosynthesis. Their study also suggests the possibility of parallel biosynthetic pathways for acteoside. The downstream pathway from caffeoyl-CoA, salidroside, and hydroxytyrosol to acteoside involves hydroxylation, glycosylation, and acylation and these are important modification reactions for the skeleton formation of phenylethanoid glycoside.

Recent reports on modification reactions in the downstream pathway are indicated by red, blue and green arrows in Figure 4. Fuji et al. (2023) performed transcriptome analysis of methyl jasmonate (MeJA)-treated sesame cultured cells to identify enzyme genes responsible for glucosylation and acylation in the acteoside biosynthetic pathway (shown as red arrows in Figure 4). Enzyme assays using recombinant UGT proteins from *S. indicum* revealed that UGT85AF10 and UGT85AF11 exhibited glucosyltransferase activity against hydroxytyrosol and tyrosol to produce hydroxytyrosol 1-*O*-glucoside and salidroside (tyrosol 1-*O*-glucoside), respectively. Additionally, enzyme assays using the recombinant SiATs indicated that SiAT1 and SiAT2 were able to transfer the caffeoyl group to hydroxytyrosol 1-*O*-glucoside and salidroside (tyrosol 1-*O*-glucoside), but not to decaffeoylacteoside. Based on these results, the authors propose an acteoside biosynthetic pathway induced by MeJA treatment in sesame (Figure 4). On the other hand, Yang et al. (2023) proposed an acteoside biosynthetic pathway in *L. robustum* (shown as blue arrows in Figure 4). They reported that osmanthuside B, the major component of *L. robustum*, is synthesized from salidroside, *p*-coumaroyl-CoA and rhamnose, and that acteoside is synthesized via the hydroxylation of osmanthuside B by the CYP98A family as the final step. However, Saimaru and Orihara (2010) reported that acteoside in olive cultured cells was biosynthesized from tyrosine via dopamine. That is hydroxylation occurs in the pathway from tyrosine to dopamine before glycosylation, and acteoside is not formed through hydroxylation of salidroside. While osmanthuside B has been detected in lemon verbena (Sanchez-Marzo et al. 2019) and cistanche herb (Deyama et al. 2006), it has not been detected in sesame and olive, which is in the same Oleaceae family as *L. robustum*, suggesting that the biosynthetic pathway of acteoside may differ among plant species. This hypothesis is also supported by the report on OfPPO2 by Liu et al. (2024). OfPPO2 has broad substrate specificity to hydroxylate a variety of monophenolic substrates and is also involved in the formation of catechol structures in the downstream intermediates osmanthuside A and B. These findings suggest that OfPPO2, along with other enzymes (UGT and AT, etc.), may contribute to the production of acteoside through multiple parallel pathways.

Other PhGs differ from acteoside in the binding positions of caffeic acid and rhamnose, or acteoside has further glycosylated (glucose, rhamnose, apiose, etc.) or acetylated structures. For instance, the biosynthetic pathways of forsythoside A, which rhamnose binding position differs from acteoside, and echinacoside, which is further glucosylated from acteoside, remain unclear. Candidate genes were selected by transcriptome analysis of *Forsythia* species (*F. suspensa*, *F. viridissima* and *F. koreana*) (Li et al. 2023; Sun et al. 2018; Yuan et al. 2022). However, functional analysis of these candidate genes has not been conducted.

As described above, the studies on acteoside biosynthesis that have been carried out over a long period of time are largely elucidated. However, it should be noted that recent reports on the biosynthesis of osmanthuside B and acteoside (Fuji et al. 2023; Liu et al. 2024; Yang et al. 2023) are primarily based on gene cloning and in vitro functional analysis using heterologously expressed enzymes. It remains unknown whether they are truly involved in PhG biosynthesis in planta. The elucidation of the PhG biosynthetic mechanism is anticipated through genetic functional studies using transgenic organisms.

## Biotechnological production and (bio)synthesis of acteoside

The low content of PhGs in most plant species has limited an investigation of their activities and industrial applications (Alipieva et al. 2014). It is important to develop sustainable methods to produce valuable acteoside for pharmaceutical applications. The extraction and purification of natural products from plants are typically laborious processes with low yields (Alipieva et al. 2014; Courdavault et al. 2021; Cravens et al. 2019). Thus, because of the diverse biological activities of PhGs, the total synthesis of PhGs has received significant attention from synthetic chemists. To date, a range of naturally occurring PhGs have been synthesized. These chemical syntheses are being actively investigated not only for acteosides but also for other PhGs (Kawada et al. 2006; Liu et al. 2015; Narayanan et al. 2023). However, chemical synthesis is low-yielding and requires multiple complex steps (Duynstee et al. 1999; Kawada et al. 1999, 2002).

The cell or tissue cultures of some plant species have been established to produce high-value compounds. In vitro systems using elicitor compounds that activate plant’s defense strategies to enhance specialized metabolite production have also been employed (Narayani and Srivastava 2017). As described below, several studies have demonstrated that both non-biological and biological elicitors can induce PhGs formation by stimulating metabolic networks. Studies utilizing various elicitors have been conducted on cell suspension cultures of *Cistanche* species (*C. deserticola*, *C. salsa*, and *C. tubulosa*). Various elicitors, such as chitosan (Cheng et al. 2006), fungal elicitors (Lu and Mei 2003), yeast elicitors (Cheng et al. 2005), putrescine (Chen et al. 2007), Ag^+^ (Chen et al. 2007), MeJA (Liu X et al. 2018), and salicylic acid (SA) (Liu X et al. 2018), have been found effective in inducing acteoside and echinacoside production. In each case, an increase in phenylalanine ammonia-lyase activity through elicitation has been observed. MeJA and SA have also been found effective in inducing acteoside production in *R. glutinosa*, *Clerodendrum indicum*, and *Acanthus ebracteatus* (Boonsnongcheep et al. 2021; Rahmat et al. 2021; Wang F et al. 2017). Rahmat et al. (2021) have developed a scaled-up production system using *R. glutinosa* adventitious root cultures and bioreactors. When MeJA was applied at a concentration of 200 µM, the biosynthesis of acteoside was most enhanced, showing a 2.53-fold increase (14.37 mg g^−1^ DW) over the control (5.67 mg g^−1^ DW) (Rahmat et al. 2021). In in vitro cultures of *C. indicum* and *A. ebracteatus*, the production of acteoside increased to 31.41±3.10 mg g^−1^ DW (4.2 times higher than the control) and 51.44±0.10 mg g^−1^ DW (3.2 times higher than the control), respectively, through the induction by MeJA. MeJA and SA are well-known signal transduction compounds that induce systemic acquired resistance in plants, in response to various pathogens, and can trigger the accumulation of specific metabolites. The application of exogenous MeJA and SA has been shown to induce physiological activities comparable to endogenous responses, such as oxidative stress and hypersensitivity, and likely promote the accumulation of bioactive compounds (Narayani and Srivastava 2017; Singh and Dwivedi 2018). Thus, MeJA and SA may effectively induce the production of acteoside and other PhGs in various plants.

*Escherichia coli* and *Saccharomyces cerevisiae*, as model microorganisms, are good hosts for the production of plant specialized metabolites. The heterologous assembly of natural product biosynthetic pathways is achieved by introducing or reassembling enzymes from different organisms or pathways into microorganisms. Large-scale production can be realized by taking advantage of the simple cell structure and rapid growth of microorganisms. The efficient synthesis of caffeic acid, hydroxytyrosol, tyrosol, and salidroside, as intermediates of acteoside, using microorganisms has already been reported. Chung et al. (2017) used *E. coli* to synthesize three phenylethanoids, tyrosol, hydroxytyrosol, and salidroside. To synthesize tyrosol, aromatic aldehyde synthase (AAS) was expressed in *E. coli*. Hydroxytyrosol was synthesized using *E. coli* harboring AAS and tyrosine hydroxylase. In order to synthesize salidroside, UGT85A1 from *Arabidopsis thaliana* was found to convert tyrosol to salidroside. *E. coli* harboring AAS and UGT85A1 synthesized salidroside. Through the optimization of these three *E. coli* strains, they were able to synthesize 531 mg l^−1^ tyrosol, 208  mg l^−1^ hydroxytyrosol, and 288  mg l^−1^ salidroside, respectively.

Additionally, the recent elucidation of the acteoside biosynthetic pathway in plants (Yang et al. 2023) has made possible the heterologous production of acteoside in *E. coli*. Yang et al. (2023) constructed the biosynthetic pathway of osmanthuside B in *E. coli* and attempted the production of acteoside. The *E. coli* strains transfected with several PhG biosynthesis-related genes, including UGT85AF8 from *L. robustum*, UGT79G15 from *R. glutinosa*, and modified CYP98A20 from *S. indicum*, were cultured in a medium containing 0.2 mM *p*-coumaric acid, and they successfully produced 0.34 mg l^−1^ of acteoside. Furthermore, the addition of 5-aminolevulinic acid, a precursor of heme biosynthesis, to the medium to increase heme as a coenzyme of CYP98A20 during fermentation, increased acteoside to 2.32 mg l^−1^. However, it appears that the system for producing acteoside from glucose without the administration of precursors did not yield acteoside. The authors speculated that overexpression of several transferred genes imposed an excessive metabolic burden on the engineered strain. Discussion regarding whether acteoside production level is high or low is challenging, but the elucidation of the biosynthetic pathway has made it possible to achieve production through metabolic engineering approaches that were previously difficult. This represents a remarkable advancement in the biomanufacturing of PhGs.

In addition, the fine-tuning of metabolic pathways and optimization of production processes are required to improve productivity. These obstacles will be overcome in the near future, and the industrial production of some phenylethanoids using plant and/or microbial systems will be possible.

## Conclusion and perspectives

The study on the molecular mechanism of natural product biosynthesis is one of the hot spots in the current specialized metabolism in medicinal plants. There have been many developments in the biosynthetic pathway of the typical PhGs, acteoside, and metabolic engineering strategy for the production of several important intermediates in acteoside biosynthetic pathway including tyrosol, hydroxytyrosol and salidroside. Although the biosynthetic pathway of acteoside has been studied and is approaching complete elucidation, there is little understanding of the coordination of key enzymes in the acteoside biosynthetic pathway and little research has been conducted on the regulatory factors of related genes. To truly understand the mechanism of acteoside biosynthesis, the technical development for gene overexpression, gene repression, and gene editing in various acteoside-producing plant species is necessary to examine the role of candidate genes responsible for acteoside biosynthesis.

The application of multi-omics strategies involving genomics, transcriptomics, proteomics, and metabolomics and other new technologies such as genome editing will provide a powerful means for fully elucidating the biosynthetic pathways and regulatory mechanisms. Based on these understanding, we expect improvements the acteoside production in engineered microorganisms and plants by optimizing the metabolic fluxes and enhancing the expression of relevant genes through engineered biological approaches.

## Abbreviations

AT: acyltransferase
PhGs: phenylethanoid glycosides
UGT: UDP-dependent glycosyltransferases
